# Supplementary material for: Inflammatory Biomarkers for Thrombotic Risk Assessment in Multiple Myeloma Patients on IMiD/aCD38-Based Regimens: Insights from a Prospective Observational Study
Source: Biomolecules. 2025 Oct 31;15(11):1533. doi: 10.3390/biom15111533 (PMC12650601; doi:10.3390/biom15111533)
Supplement: Supplementary file 1 [file biomolecules-15-01533-s001.zip › biomolecules-3877456-supplementary.pdf]

biomolecules

MDPI

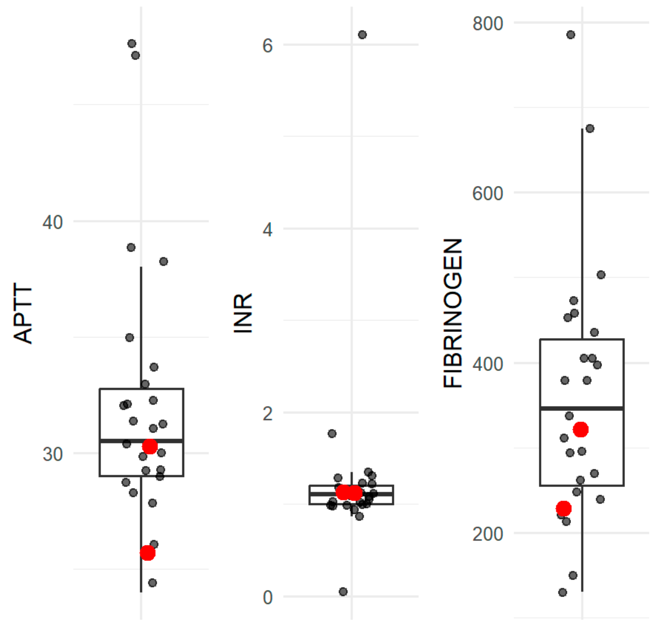

Figure S1: "First level" coagulation assays. Boxplot representing data of first level coagulation tests; in black MM patients not experiencing VTE, while in red MM patients who experienced VTE.

Table S1: patient's risk scores

PATIENT	IMWG	IMPEDE	SAVED	PRISM	THROMBOSIS
PT1	High Risk	intermediate risk (6)	Low Risk	UNK	NO
PT2	High Risk	intermediate risk (7)	Low Risk	UNK	NO
PT3	High Risk	intermediate risk (4)	Low Risk	UNK	NO
PT4	High Risk	low risk (3)	Low Risk	UNK	NO
PT5	High Risk	intermediate risk (7)	Low Risk	UNK	YES
PT6	High Risk	low risk (3)	Low Risk	UNK	NO
PT7	High Risk	High risk (10)	High Risk	UNK	NO
PT8	High Risk	intermediate risk (7)	Low Risk	UNK	NO
PT9	High Risk	High risk (8)	UNK	UNK	NO
PT10	High Risk	Intermediate risk (7)	Low Risk	UNK	NO
PT11	High Risk	Intermediate risk (7)	Low Risk	UNK	NO
PT12	High Risk	Low risk (0)	UNK	UNK	NO
PT13	UNK	UNK	UNK	UNK	NO

PATIENT	IMWG	IMPEDE	SAVED	PRISM	THROMBOSIS
PT14	High Risk	Low risk (3)	Low Risk	UNK	NO
PT15	High Risk	Low risk (2)	UNK	UNK	NO
PT16	High Risk	Low risk (2)	UNK	UNK	NO
PT17	High Risk	Low risk (-1)	UNK	UNK	NO
PT18	High Risk	Low risk (3)	High Risk	UNK	NO
PT19	High Risk	UNK	UNK	UNK	NO
PT20	High Risk	Intermediate risk (7)	Low Risk	UNK	YES
PT21	UNK	UNK	UNK	UNK	NO
PT22	High Risk	Low risk (3)	Low Risk	UNK	NO
PT23	High Risk	Intermediate (4)	UNK	UNK	YES
PT24	High Risk	High risk (8)	High Risk	UNK	NO
PT25	High Risk	Intermediate risk (4)	UNK	UNK	NO
PT26	High Risk	Intermediate risk (6)	High Risk	UNK	NO
PT27	UNK	UNK	UNK	UNK	NO
PT28	High Risk	Intermediate risk (6)	Low Risk	UNK	NO
PT29	High Risk	Intermediate risk (6)	Low Risk	UNK	NO
PT30	High Risk	Low risk (-1)	UNK	UNK	NO
PT31	High Risk	Low risk (3)	UNK	UNK	NO
PT32	High Risk	Intermediate risk (6)	High Risk	UNK	NO
PT33	High Risk	Intermediate risk (6)	Low Risk	UNK	NO
PT34	High Risk	Intermediate risk (6)	Low Risk	UNK	YES
PT35	High Risk	Intermediate risk (6)	High risk	UNK	NO
PT36	High Risk	low risk (-1)	UNK	UNK	NO
PT37	High Risk	intermediate risk (4)	Low Risk	UNK	NO
PT38	High Risk	intermediate risk (4)	Low Risk	UNK	NO
PT39	High Risk	high risk (8)	Low Risk	UNK	NO
PT40	High Risk	high risk (8)	High Risk	UNK	NO

PATIENT	IMWG	IMPEDE	SAVED	PRISM	THROMBOSIS
PT41	High Risk	intermediate risk (7)	Low Risk	UNK	NO
PT42	High Risk	Intermediate risk (4)	Low Risk	UNK	NO
PT43	UNK	UNK	UNK	UNK	NO
PT44	High Risk	intermediate risk (7)	Low Risk	UNK	NO
PT45	High Risk	intermediate risk (6)	Low Risk	UNK	YES
PT46	High Risk	intermediate risk (7)	High Risk	UNK	NO
PT47	High Risk	intermediate risk (6)	High Risk	UNK	NO
PT48	High Risk	intermediate risk (6)	Low Risk	UNK	NO
PT49	High Risk	intermediate risk (6)	Low Risk	UNK	NO
PT50	High Risk	intermediate risk (7)	Low Risk	UNK	NO
PT51	High Risk	intermediate risk (7)	Low Risk	UNK	NO
PT52	High Risk	intermediate risk (7)	Low Risk	UNK	NO
PT53	UNK	UNK	UNK	UNK	NO
PT54	High Risk	intermediate risk (7)	low risk	UNK	NO
